# Supplementary material for: Stacking Model‐Based Classifiers for Dealing With Multiple Sets of Noisy Labels
Source: Biom J. 2025 Mar 12;67(2):e70042. doi: 10.1002/bimj.70042 (PMC11898607; doi:10.1002/bimj.70042)
Supplement: Supplementary file 1 — Supporting Information [file BIMJ-67-e70042-s001.zip › density-based-ensemble-model-main/competitors_real_data/netlab-master/netlab-master/help/demard.htm]

Netlab Reference Manual demard


# demard

## Purpose

Automatic relevance determination using the MLP.

## Synopsis

```
demmlp1
```

## Description

This script demonstrates the technique of automatic relevance
determination (ARD) using a synthetic problem having three input
variables: `x1` is sampled uniformly from the range (0,1) and has
a low level of added Gaussian noise, `x2` is a copy of `x1`
with a higher level of added noise, and `x3` is sampled randomly
from a Gaussian distribution. The single target variable is determined
by `sin(2*pi*x1)` with additive Gaussian noise. Thus `x1` is
very relevant for determining the target value, `x2` is of some
relevance, while `x3` is irrelevant. The prior over weights is
given by the ARD Gaussian prior with a separate hyper-parameter for
the group of weights associated with each input. A multi-layer
perceptron is trained on this data, with re-estimation of the
hyper-parameters using `evidence`. The final values for the
hyper-parameters reflect the relative importance of the three inputs.

## See Also

`demmlp1`, `demev1`, `mlp`, `evidence`

---

**Pages:**
Index


---

Copyright (c) Ian T Nabney (1996-9)
